# Origin of carbonatites—liquid immiscibility caught in the act

**DOI:** 10.1038/s41467-022-30500-7

**Published:** 2022-05-24

**Authors:** Jasper Berndt, Stephan Klemme

**Affiliations:** Institut für Mineralogie, Westfälische Wilhelms-Universität Münster, Corrensstraße 24, 48149 Münster, Germany

**Keywords:** Petrology, Geochemistry

## Abstract

Carbonatites are rare but worldwide occurring igneous rocks and their genesis remains enigmatic. Field studies show a close spatial but controversially debated genetic relationship with alkaline silicate rocks, and petrological and experimental studies indicate liquid immiscibility from mantle-derived magmas being one viable model for the generation of carbonatites. However, unaltered carbonatitic melts are rare and the composition of primary carbonate liquids and their silicate conjugates is poorly constrained. Here we show an example of primary Ca-carbonatitic melt formed by liquid immiscibility from a phonolitic magma of the Laacher See volcano (Eifel, Germany). The conjugate blebs of carbonate-silicate liquids are found in hauyne-hosted melt inclusions. The Ca-carbonatite melts are moderately alkali-rich and contain high F and Cl at elevated SiO_2_ and Al_2_O_3_ concentrations. Such carbonatite liquids are viable parental magmas to the globally dominating intrusive Ca-carbonatite complexes and may provide the missing link to extrusive Na-carbonatitic magmas.

## Abstract (web summary)

Carbonatites are fascinating igneous rocks and their genesis remains enigmatic. Here the authors show that a Ca-rich carbonatite melt formed by liquid immiscibility from a phonolitic magma of the Laacher See volcano in the Eifel, Germany.

## Introduction

Carbonatites are igneous rocks with significant amounts of magmatic carbonate (>50 vol%^1^), only little silica, and their origin remains uncertain. Field observations often show a close spatial relationship with alkaline silicate rocks^2^. However, the genetic link between carbonate and silicate rocks^3,4^ as well as the petrological evolution of carbonatite melts has been a matter of debate for decades (e.g.^5,6^) and several hypothesis have been proposed. These models include extreme differentiation by fractional crystallization (e.g.^7,8^) and/or separation of a carbonatite from an immiscible (e.g.^9–15^) CO_2_-rich parental silicate melt or primary mantle-derived carbonate melts (e.g.^16,17^).

Many carbonatites associated with silica-undersaturated alkaline rocks are thought to form by liquid immiscibility from mantle-derived alkaline silicate magmas, as proposed from field evidence for numerous locations e.g. (Oldoinyo Lengai^18–20^, Kerimasi^21–23^, Shombole^24^, Grønnedal-Ika^25^, Gardiner complex^26^).

In order to asses the nature of the physical processes and petrogenetic relationships leading to carbonatites, genuine bulk compositions of primary carbonatite would be required, as well as the textural setting in which they may occur with conjugating alkaline silicates. Such knowledge could also contribute to the longstanding question why the majority of carbonatitic rocks worldwide are Ca-carbonatites while the only active carbonatite volcano (Oldoinyo Lengai, East African Rift, Tanzania) is Na-carbonatitic^27^.

As most carbonatites are plutonic^28^, usually coarse grained and mostly cumulates from carbonatitic magmas, such rocks cannot represent primary carbonate quenched liquids^5^ and clear textural evidence of petrogenetic processes such as liquid immiscibility is largely lost with some notable exceptions (e.g.,^29,30^). Therefore, extrusive carbonatites may be better suited to determine primary carbonatite melt compositions^31^, but the latter are rare (∼10% of known carbonatite occurrences worldwide^31^) and often heavily altered.

Melt inclusions offer a better way to understand the petrogenesis of carbonatites, as demonstrated by numerous studies (e.g.,^20–22,29,32–36^) indicating liquid immiscibility is a key process in the formation of carbonatites. However, data from unaltered consanguine silicate–carbonate liquids are rare as many carbonate-silicate melt inclusions underwent at least partial recrystallization and/or alteration.

Brooker and Kjarsgaard^9^ showed experimentally that low to moderately alkaline carbonatites can be produced at crustal pressures by liquid immiscibility from a silica-undersaturated alkaline-magma. Such carbonate liquids contain ~5 wt% Na_2_O + K_2_O, significant amounts of SiO_2_ + Al_2_O_3_ (>10 wt%), and may be parental to common Ca-carbonatites (e.g.,^9,37^) by accumulation and fractionation processes. However, such moderately alkaline Ca-carbonatite liquids with significant amounts of SiO_2_ + Al_2_O_3_ have not been found in nature, yet.

Here we present results from a study of hauyne-hosted conjugate silicate–carbonate melt inclusions from the phonolitic Laacher See volcano (13,006 ± 9 years BP^38^), located in the alkaline continental intraplate East Eifel Volcanic Field, Germany. The crystal-free liquids are rapidly quenched by the volcanic eruption and due to their young age unaltered. Furthermore, the pre-eruptive conditions of the Laacher See volcanic system are well investigated allowing accurate constraints of pressure, temperature, and compositional parameters at which silicate–carbonate liquid immiscibility in mantle-derived alkaline magmas can take place, thus allowing a new insight into the origin of carbonatites.

## Results

### Geological setting

The Laacher See volcano erupted 13,006 ± 9 years BP (BP as AD 1950)^38^ in less than 10 days^39^ about 5.3 km^3^ phonolitic magma, with eruption types alternating mainly between phreatomagmatic activity and plinian eruptions^40,41^. The Laacher See Tephra (LST) deposits are well preserved and allow a detailed reconstruction of magma chamber conditions. The single magma reservoir was chemically and mineralogically zoned, ranging from crystal-rich mafic phonolites at the bottom towards highly differentiated, volatile-rich and phenocryst-poor phonolites at the top^40^. Temperatures varied from 880 °C in the lower parts to 720 °C in the uppermost section^41,42^. The depth of the magma chamber is estimated at 3–6 km with pressures between 100 and 200 MPa^42,43^. The basanitic parental magma of the LST differentiated over 100 kyr in the deeper crust to a mafic phonolitic melt, during which it continuously fractionated and ascended into shallow crustal levels^41,44^. U-Th zircon ages^44,45^ indicate that a highly evolved phonolitic magma had already existed 10–20 kyr prior eruption.

Laacher See phonolites are silica-rich (54.4–58.2 wt% SiO_2_), Mg-poor (0.09–1.1 wt% MgO) and are characterized by high alkali concentrations (11.6–17.3 wt% Na_2_O + K_2_O)^40^. The most important phenocrysts are sanidine, plagioclase, hauyne, amphibole, clinopyroxene, titanite, magnetite, phlogopite, apatite, and zircon. The LST is high in incompatible trace elements^40^ and volatiles like F (690–4060 ppm), Cl (1770–4400 ppm), and S (150–1490 ppm)^46^. Melt inclusions occur in all phenocrysts present in the LST^47^ and are not significantly modified after entrapment^46^. Crustal contamination or secondary alteration of the LST in general are negligible as constrained from strontium- and oxygen isotope studies^48,49^.

Carbonatitic syenites, enclosing the Laacher See magma chamber as a mostly crystalline carapace^50,51^, occur as clasts in the middle and late erupted LST, and are consanguine to Laacher See phonolite magma as concluded from conjugate phonolite—carbonatite trace element patterns^50,52^ as well as carbon- and oxygen isotopes^45,52^. While the exact mechanism of carbonatite melt formation remains unclear^52^, Schmitt et al.^45^ and Rout and Wörner^50^ suggest liquid immiscibility between carbonatite and phonolite liquids with subsequent fast segregation of the carbonatite melt forming a carbonatite-syenite intrusive complex at the magma chamber margin^45^. Three different groups of the Laacher See Carbonatites (LSC) can be distinguished^45,52^: LSC 1, which is a nosean-syenite with sövite droplets indicating liquid immiscibility. LSC 2 is a hybrid sövite-syenite that may have formed by either remixing of carbonatite and syenite or represent co-crystallized conjugate silicate and carbonate melts that were not completely separated after unmixing. LSC 3 is a residual calcite-bearing nosean-syenite. Major components of the LSC are calcite, nosean, and sanidine as well as less abundant clinopyroxene, albite, and garnet. Accessory phases are magnetite, biotite, zircon, apatite, and pyrochlore. Rhodochrosite, cancrinite, allanite, and fluorite occur sporadically^45,52^. Overall, the association of alkaline silicate rocks with Ca-carbonatites in the LST is typical, albeit at larger scales, for many intrusive carbonatite complexes worldwide (e.g.^53^).

### Immiscible carbonate-silicate liquid compositions

**Fig. 1:**
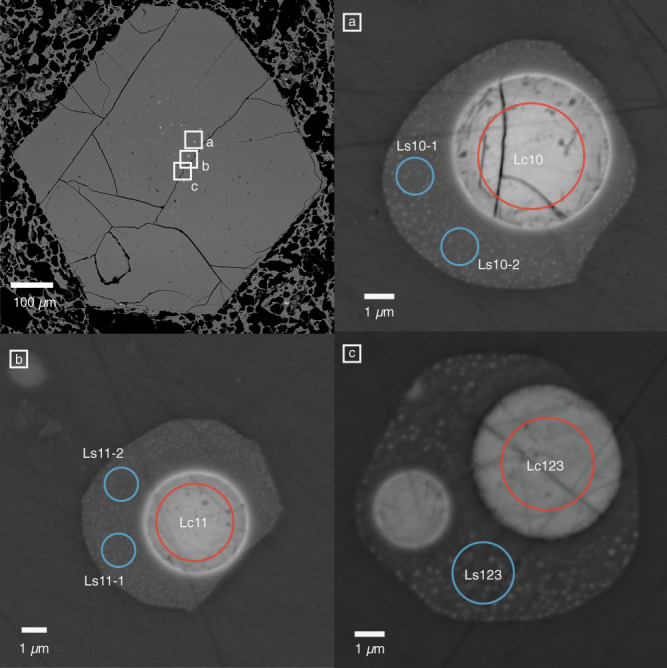
Conjugate immiscible silicate–carbonate liquids. Back-scattered electron (BSE) images of euhedral hauyne host crystal embedded in phonolitic pumice lapilli from the Laacher See volcano showing melt inclusions (**a**–**c**) with conjugate immiscible silicate (Ls) and carbonatite (Lc) liquids. Red and blue circles mark EPMA analyses spots. Melt inclusions are larger in the central part of the hauyne host crystals while the outer parts are inclusion-free.

Here we show conjugate blebs of quenched carbonate-silicate liquids in hauyne-hosted melt inclusions (<1–20 µm in diameter) from the LST deposits (Fig. 1). The mm-sized euhedral hauyne crystals are embedded in highly vesicular phonolitic pumice lapilli from the middle Laacher See Tephra (MLST; Layer 1034^40^). Those hauynes might be phenocrysts, or they may have been derived from a crystal-rich carbonatitic syenite carapace that surrounded the erupted phonolite melt. The presence of such carapace-derived crystals in the main magma body has been demonstrated by^44,54,55^.

**Fig. 2:**
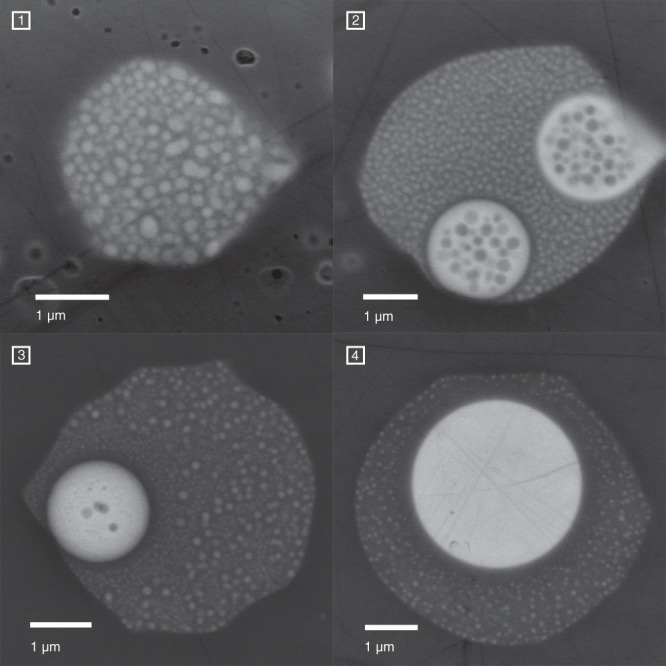
Liquid immiscibility separation stages. BSE images of melt inclusions showing different separation stages of liquid immiscibility. **1** nuclei formation of immiscible carbonatite liquids (Lc) in silicate (Ls) after the critical solution temperature of the silicate–carbonate system was crossed and the melt became metastable. **2** coalescence of primary droplets forming Lc blobs while physical separation is still poor and Ls is dispersed in Lc and vice versa. **3** further coarsening of Lc. Note that Lc droplets decrease in size and abundance in the close proximity of larger the carbonate blob. **4** formation of a homogenous carbonate melt blob. As the separation is presumably quenched by the volcanic eruption the silicate conjugate still contains finely dispersed nm-sized Lc nuggets except in the very proximity of the large carbonate blob (see also Supplementary Notes).

While most of the melt inclusions consist of phonolitic glass (Ls) only, about 5% exhibit additional globular carbonate melt droplets (Lc) (<1–5 µm). High-resolution BSE images show that the phonolitic part of those melt inclusions also contain nm-sized carbonate liquid droplets which are less abundant in the close proximity of larger carbonate blebs (Fig. 2) as a result of coarsening driven by decrease in interfacial free energy^56^. Some of the melt inclusions contain bubbles indicating the presence of a vapour phase. Modal abundance of carbonate and phonolitic melt entrapped in the melt inclusions is ∼4% and ∼96%, respectively (see “Methods” and Fig. 1 in the Supplementary Notes). 36 conjugate silicocarbonate to carbonate-silicate liquid pairs were sufficiently large enough to be analyzed with field-emission electron microprobe (EPMA) techniques. Furthermore, we measured 23 melt inclusions with only phonolitic liquids (Ls*) and 13 carbonate melts, where the conjugating phonolitic part was too small to perform quantitative analyses (Lc*). Representative compositions are given in Table 1, all microprobe analyses are presented in the Supplementary Data 1.

**Table 1:** Selected electron microprobe analyses of conjugate immiscible silicate (Ls) – carbonate (Lc) melts.

	Lc7	Ls7^‡^	Lc10	Ls10-1^‡^	Lc100	Ls100^‡^	Lc101	Ls101^‡^	Lp
		(−15% Lc7)		(−11% Lc10)		(−8% Lc100)		(−11% Lc101)	
wt%									
SiO_2_	14.88	54.61	15.3	57.19	15.58	60.48	16.30	61.65	54.30
TiO_2_	0.46	0.58	0.67	0.44	0.68	0.21	0.48	0.13	0.26
Al_2_O_3_	1.34	20.15	1.234	20.44	0.82	21.02	0.89	22.04	19.78
Cr_2_O_3_	0.079	bdl	bdl	bdl	0.13	bdl	bdl	0.24	0.04
FeO	2.31	2.11	2.34	1.73	3.04	1.40	3.16	2.10	1.68
MnO	0.553	0.37	0.731	0.30	1.40	0.508	1.05	0.058	0.2
MgO	0.741	0.117	0.565	0.03	0.49	0.07	0.32	0.11	0.33
CaO	51.61	0.69	46.78	0.36	52.39	0.69	51.58	bdl	3.83
P_2_O_5_	n.a.	n.a.	n.a.	n.a.	0.12	0.07	0.18	bdl	
Na_2_O	4.00	6.44	3.83	6.03	3.35	5.20	3.96	4.77	5.1
K2O	0.707	8.51	0.496	8.46	0.56	7.23	0.43	6.14	6.49
SO_3_	0.445	0.91	0.512	2.02	0.30	0.239	0.33	0.159	0.76
Cl	0.4005	0.199	0.4157	0.173	0.41	0.188	0.49	0.175	0.22
F	4.036	0.461	3.966	0.157	3.97	0.25	4.20	0.16	0.42
CO_2_					17.25^§^		19.04^§^	0.63	0.57
Sub total	81.56	95.15	76.84	97.34	100.48	97.56	102.42	98.35	94.01
Less O = F,Cl	1.79	0.24	1.76	0.11	1.76	0.15	1.88	0.11	0.23
Total	79.77	94.91	75.08	97.23	98.72	97.41	100.54	98.25	93.84
CO_2_^†^	20.23		24.92						

Lp represents the calculated parental phonolite melt. All Ls-Lc EPMA analyses are shown in Supplementary Data 1.

*bdl* below detection limit, *n.a.* not analysed.

^†^ CO_2_ in carbonate liquid estimated by difference to 100 wt% total.

^§^ CO_2_ with C measured quantitatively.

‡ Ls composition corrected for coalescing nm-sized Lc melt droplet component. Modal % Lc in Ls in parantheses.

**Fig. 3:**
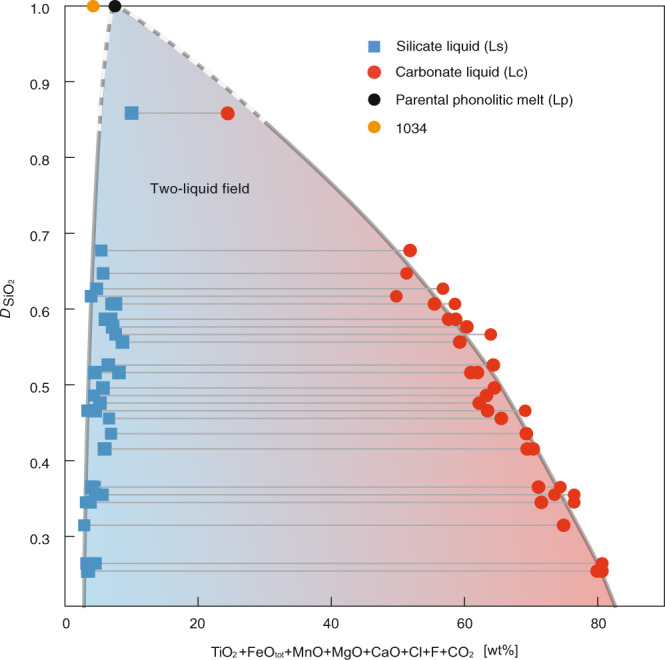
Compositions of conjugate immiscible carbonate and silicate melt. Conjugate immiscible carbonate and silicate melt compositions plotted as a function of the partition coefficient of SiO_2_ between carbonate and silicate melt ($D_{{{{\mathrm{SiO}}}}_{2}}$) showing the compositional gap between the two melts. Calculated parental phonolitic melt (Lp) is given in Table 1. LST layer 1034 bulk rock composition is taken from^40^.

The compositional trend of conjugate immiscible melts indicates a two-liquid field (Fig. 3). The two-liquid field expands depending on the degree of separation of the immiscible carbonate-silicate melts, while the carbonatitic melts show a wider compositional range than conjugating silicate liquids. The silicocarbonatite to carbonatite liquids have Na_2_O + K_2_O concentrations ranging from 1.7–7.8 wt% (average 4.2 wt%), SiO_2_ + Al_2_O_3_ vary between 16.2–68.8 wt%. CaO and CO_2_ range from 12.7–52.4 and 5.4–24.9 wt%, respectively. Consanguine phonolite melts show SiO_2_ + Al_2_O_3_ concentrations of 72.8–83.7 wt%, CaO of up to 5.5 wt% and Na_2_O + K_2_O varying between 10.3–15 wt%.

**Fig. 4:**
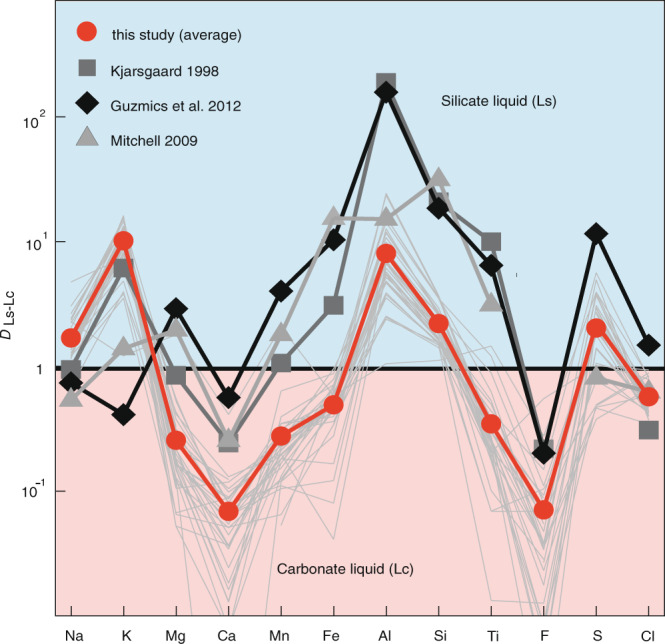
Major element distribution between immiscible silicate and carbonate liquids. Partition coefficients (*D*_Ls/Lc_) for major element oxides between immiscible silicate and carbonate liquids in hauyne-entrapped melt inclusions. Mg, Ca, Mn, Fe, F, Ti, and Cl partition into the carbonate melt while Na, K, Al, Si, S are concentrated in the silicate liquid. In general our data agrees well with experimental data from Kjarsgaard^37^ on carbonated nephelinite at 200 MPa and 900–960 °C and natural data from Guzmics et al.^22^ on heated perovskite-hosted coexisting immiscible melt inclusions from nephelinitic rocks (Kerimasi volcano) indicating that equilibrium between the immiscible melts has been attained. All Fe is shown as FeO_tot_.

The major element distribution between immiscible silicate and carbonate melts is illustrated in Fig. 4 as *D*_Ls/Lc_. Overall, the carbonate melts are enriched in Ca, Mg, Mn, Fe_tot_, Ti, F, and Cl (*D*_Ls-Lc_ < 1) while Na, K, Al, Si, and S are concentrated in the silicate liquid (*D*_Ls-Lc_ > 1).

Using these data, we calculated the parental melt composition from which Ls and Lc were formed (Table 1), using the compositions and modal abundances of Ls, Lc, and Ls* (see Supplementary Data 1). It should be noted that the phonolite liquids (Ls) as well as the calculated parental melt (Lp) are relatively rich in CaO, which indicates that a more primitive phonolite melt, which resembles melt compositions from lower parts of the magma chamber (ULST), has been entrapped.

## Discussion

The composition of primary carbonatite melts is controversially discussed as the vast majority of >500 carbonatite occurrences worldwide^57^ are calcitic or dolomitic while the only active carbonatite volcano Oldoinyo Lengai erupts natrocarbonatitic lavas^27^. Several authors (e.g.,^58,59^) proposed that Ca-Carbonatites derive from Na-carbonatitic melts and have lost their alkalis by fenitization or other fluid-driven processes. Chen et al.^32^ deduced from melt inclusions in the calciocarbonatitic Oka complex which contain i.a. nyerereite that its parental liquid was natrocarbonatitic and that such alkali-rich carbonate melts were more common than preserved in the carbonatite rock record.

**Fig. 5:**
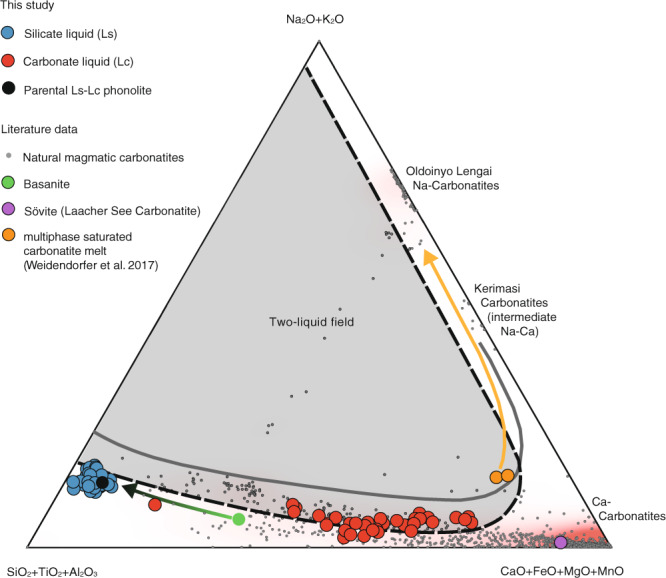
Ternary plot of natural and experimental data. Hamilton projection^70^ i.e. ternary plot with CaO + MgO + FeO vs SiO_2_ + Al_2_O_3_ + TiO_2_ vs Na_2_O + K_2_O (in wt%) of the conjugating immiscible silicate (Ls) and carbonate liquid (Lc) from this study. Also plotted: calculated parental phonolite (Table 1), Sövite (Laacher See Carbonatite^52^, and Weidendorfer et al.’s^60^ Ca-carbonatitic parental “multiphase” composition (100 MPa at 1000 and 1050 °C) with liquid line of descent^60^ (orange line). The dashed line is the estimated binodal from this study, the solid line is the binodal determined by Kjarsgaard^37^ for CaO-rich nepheline at 200 MPa. For comparison, compositions of natural magmatic carbonatites^57^ with compositions taken from the GEOROC (Geochemistry of Rocks of the Oceans and Continents) database (http://georoc.mpch-mainz.gwdg.de/georoc/) are plotted. The heat map (highlighted in red) shows relative distribution of magmatic carbonatite compositions. Also shown is the indicated differentiation path from mantle-derived basanitic (green circle) to phonolitic magma^41,44^.

Contrastingly, a recent experimental study of Weidendorfer et al.^60^ shows that Na-carbonatites can evolve from moderately alkali-rich Ca-carbonatite liquids at crustal pressures (100 MPa, 1200–590 °C) through crystal fractionation. They proposed that a parental melt (i.e., their “multiphase” composition: 8–9 wt% Na_2_O + K_2_O, Fig. 5) represent such a moderately alkali-rich Ca-carbonatite melt that could have exsolved from nephelinites, but natural examples of such melts have not been reported, yet. However, experimental findings of Kjarsgaard^37^ on CaO-rich nephelinites at 200–500 MPa and 900–1040 °C show that low to moderately alkali (4.23–17.76 wt% Na_2_O + K_2_O) and Ca-rich carbonatite melts at SiO_2_-contents between 1.11–11.72 wt% can be generated, namely by liquid immiscibility (Fig. 5).

The immiscible carbonatite melts in our melt inclusions trend towards such moderately alkali-(${\bar{D}}_{{{{{{\rm{Ls}}}}}}/{{{{{\rm{Lc}}}}}}}^{{{{{{\rm{Na}}}}}}}$= 1.8; ${\bar{D}}_{{{{{{\rm{Ls}}}}}}/{{{{{\rm{Lc}}}}}}}^{{{{{\rm{K}}}}}}$ = 10.7) and highly Ca-rich (${\bar{D}}_{{{{{{\rm{Ls}}}}}}/{{{{{\rm{Lc}}}}}}}^{{{{{{\rm{Ca}}}}}}}$ = 0.07) compositions at elevated SiO_2_ and Al_2_O_3_ contents (Figs. 4, 5). This is especially indicated by the most primitive carbonatitic liquid Lc10 in our study with SiO_2_ + Al_2_O_3_ of 16.5 wt%, CaO of 46.8 wt%, and Na_2_O + K_2_O of 4.3 wt% (Table 1). Furthermore, the preferred partitioning of F (*D*_Ls/Lc_ = 0.07) and Cl (*D*_Ls/Lc_ = 0.6) into carbonate liquid (Lc) results in high to moderate F (average 4.9 wt%) and Cl (average 0.39 wt%) contents while Mg, Mn, Fe, and Ti also have Ls-Lc D’s < 1 (Fig. 4). It should be noted that primary carbonatite melts must contain some Si, Al, Fe, Mg, F, Cl, and P as fluorite, apatite and other accessory silicates and oxide minerals are commonly observed to crystallize in carbonatite melts^5^. Presumably, the carbonatitic liquids found in this study are parental to the Ca-carbonatites occurring in the LST by segregation and fractionation^45^ after separation from the phonolitic melt. More importantly, the overall composition of these primary carbonatites (low to moderate alkalis, high CaO, significant SiO_2_ and Al_2_O_3_, high F and Cl) would be ideal parental magmas to the common intrusive calciocarbonatites^9,37^ as they further separate, fractionate, and accumulate.

Concerning the genesis of Na-carbonatites, the “multiphase” compositions of Weidendorfer et al.^60^ are compositionally close to Lc10 (Fig. 5) at somewhat lower SiO_2_ and Al_2_O_3_ concentrations. However, the separation of Ls-Lc was quenched by the volcanic eruption and further separation at lower temperatures would produce less SiO_2_ and Al_2_O_3_ and more alkali-rich carbonatite compositions (Fig. 5) approaching the multiphase compositions of Weidendorfer et al.^60^. Thus, their conclusion that Na-carbonatite and Ca-carbonatite rocks may have similar, moderately alkaline Ca-carbonatitic parental melts is corroborated by this study.

In conclusion, our data confirms that carbonatitic melts can be formed by liquid immiscibility from an alkaline, silica-undersaturated, highly-differentiated phonolite magma under crustal pressures and temperatures. These primary carbonatite liquids are of moderately alkaline Ca-carbonatitic compositions with significant amounts of silica, and they are ideal parental melts to the common intrusive Ca-Carbonatites (e.g.,^53,61^) but may also fractionate towards Na-rich carbonatites such as those found at the Oldoinyo Lengai volcano^60^.

## Methods

### Electron microprobe

Quantitative analyses of carbonatite and silicate melt inclusions were done with a JEOL JXA 8530F field-emission electron microprobe. Prior to analyses all elements were standardized on matrix-matched natural and synthetic reference materials (Table 2). Acceleration voltage was set to 15 kV. Given the small size of carbonate and silicate melts, in a second analytical session the accelerating voltage was set to 10 kV in order to decrease the beam interaction volume. The electron beam size was adjusted depending on size of the melt inclusions between 1 and 10 µm. Firstly, all elements except F and Cl were analyzed with a beam current of 10 nA and counting times of 10 s on the peak and 5 s on the background except for Na and K, which have been measured with 5 s on the peak and 2.5 s on the background within the first round of elements to avoid migration of alkalis. Secondly, the same spots were measured for F and Cl. Due to the interference of FeLα with FKα on high intensity LDE1 multilayer diffraction crystal we followed the procedure of Flemetakis et al.^62^ by estimating the FeLα contribution on the FKα signal using a series of F-free glasses with varying Fe-contents. Subsequently all measured F concentrations were corrected depending on the previously determined sample Fe concentrations. In a last step both analyses were merged using the Offline-matrix correction provided by the JEOL instrument software.

**Table 2:** Electron microprobe analytical conditions at 15 and 10 kV and 1–10 µm spot size.

Element	Channel	Diff. crystal	X-ray line	Beam current	Peak/Bkg. counting time (s)	Reference material
F	1	LDE1	Kα	15	30/15	Ast_Topas
C	1	LDE2	Kα	15	10/5	P_Fe3C
Na	2	TAP	Kα	10	5/2.5	H_Jadeit
Mg	2	TAP	Kα	10	10/5	U_OlivineSanCarlos
Al	2	TAP	Kα	10	10/5	H_DistheneR8
Si	2	PETJ	Kα	10	10/5	U_Hypersthene
K	3	PETJ	Kα	10	5/2.5	H_SanidineP14
Ca	3	PETJ	Kα	10	10/5	H_DiopsideST48
Cl	4	PETJ	Kα	15	30/15	Ast_Tugtupite
S	4	PETJ	Kα	10	10/5	Ast_Celestite
P	4	LIFH	Kα	10	10/5	U_Apatite_P
Ti	5	LIFH	Kα	10	10/5	Ast_Rutile
Fe	5	LIFH	Kα	10	10/5	U_Fayalite
Cr	5	LIFH	Kα	10	10/5	Ast_Cr2O3
Mn	5	LIFH	Kα	10	10/5	Ast_Rhodonite

For analyses #Lc/Ls21-32/100-124, Ls70*-75*, and Lc42*-45* carbon has been measured quantitatively using a LDE2 multilayer diffraction crystal. After careful chemical and plasma cleaning to reduce surface hydrocarbon contamination, samples and standards were sputtered with Ir. Furthermore, all specimens were routinely treated by a plasma cleaner directly connected to the sample exchange chamber of the electron microprobe before putting them into the vacuum chamber. A liquid nitrogen trap attached to the microprobe was also used to decrease C contamination. Despite all measures taken, the build-up of C contamination during analyses is not completely unavoidable. Thus, standard and sample analyses were corrected for C blank signal determined beforehand by measuring sets of pure Fe and C-bearing references steels as well as C-free and C-bearing reference silicate glasses, respectively. Linear fits of the obtained C count rates give a C blank intensity at 15 nA of 38 cps on the primary Fe_3_C carbon standard and 22 cps on silicate glasses. The C blank on carbonate melts could not be determined due to the lack of proper reference materials but is assumed to be in the range of that of silicates. Since the position and shape of C X-ray emissions change depending on the chemical bonding^63^, the position of the CKα peak was carefully determined for Fe_3_C standards and samples and varied by 0.00204 sinθ.

### Modal abundance of phonolite (Ls) and carbonate (Lc) melt

In order to determine the proportion of carbonate melt that has separated from the phonolite liquid high resolution BSE images were taken of the hauyne host crystals that allow the discrimination between the carbonate and silicate parts of the melt inclusions. Using the ImageJ software^64^ modal average proportions of immiscible phases were estimated to be ∼ 4% Lc and ∼ 96% Ls. As the particular sections through the samples are random, this approach assumes that all trapped melt inclusions in the hauyne crystals investigated represent the host melt that is parental to the conjugating immiscible Lc–Ls pairs and that the Lc–Ls proportions exposed by the hauyne sample surface are representative. Supplementary Notes Fig. 1 shows an exemplary hauyne (a) grain with modal % Ls and Lc (b).

### Correction of phonolite melt composition

The conjugated silicate melts (Ls) contain nm-sized Lc droplets (Fig. 1a–c) that cannot be avoided analyzing Ls with electron microprobe. Thus, the individual modal% Lc in Ls were determined for Lc–Lc pairs by image phase analysis with ImageJ^64^ (Table 1 and Supplementary Data 1; Conjugate immiscible melt composition). In some cases, image phase analysis was not possible due to small picture sizes, electron beam burn marks, and cracks. Those Ls compositions have been corrected using the average modal% of Lc in Ls of 15%. All Ls compositions were corrected applying simple mass balance calculations:

1 $${{{{{{\rm{Ls}}}}}}}_{i}^{{{\ddagger}} }=\frac{{{{{{{\rm{Ls}}}}}}}_{i}^{{\prime} }-\left({{{{{{\rm{Lc}}}}}}}_{i}{\omega }_{{{{{{\rm{Lc}}}}}}}\right)}{{\omega }_{{{{{{\rm{Ls}}}}}}}}$$

where Ls^‡^ is the Lc-corrected Ls composition, ω_Ls_ and ω_Ls_ are the weight fractions of the silicate and carbonate liquid, i is the respective element, and Ls’ is the silicate melt composition as analyzed by electron microprobe. Wolff et al.^65^ reported densities of hydrous Laacher See phonolites between 2.26 and 2.53 g/cm^3^ at 1 kbar and 880–800 °C. Tait et al.^66^ calculated for middle and lower Laacher See phonolites densities of ∼2.3–2.32 g/cm^3^ at 860 °C/3.5 wt% H_2_O and 875 °C/2.5 wt% H_2_O, respectively. Nesbitt and Kelly^67^ estimated a density of 2.2–2.3 g/cm^3^ for a Ca-Carbonatite magma (Magnet Cove Complex of central Arkansas) at 450 bar and 800–1000 °C. An experimental study of Ritter et al.^68^ showed densities for hydrous CaMg(CO_3_)_2_ at 2 kbar and ∼830 °C of ∼2.3 g/cm^3^ and Wolff^69^ reported densities of 2.3–2.4 g/cm^3^ at 800 °C for water-poor Ca-rich carbonate liquids while hydrous carbonatite melts may be less dense. Although Lc is most likely less dense than Ls the reported melt densities of Ca-rich carbonatites are within the density range of phonolite magmas. Thus, no further density correction converting from modal to weight proportion using equation [1] has been applied.

## Supplementary information

Supplementary Information

Description of Additional Supplementary Files

Supplementary Data 1

## Data Availability

The data supporting the findings of this study are provided as Supplementary Data 1 within the paper.
