# Supplementary material for: Origin of carbonatites—liquid immiscibility caught in the act
Source: Nat Commun. 2022 May 24;13:2892. doi: 10.1038/s41467-022-30500-7 (PMC9130134; doi:10.1038/s41467-022-30500-7)
Supplement: Supplementary file 1 — Supplementary Information [file 41467_2022_30500_MOESM1_ESM.pdf]

**Origin of Carbonatites – Liquid Immiscibility Caught in the Act**

Jasper Berndt[1],* & Stephan Klemme[1]

[1] Institut für Mineralogie, Westfälische Wilhelms-Universität Münster, Corrensstraße 24, 48149 Münster

Email: jberndt@uni-muenster.de, stephan.klemme@uni-muenster.de

*Corresponding author

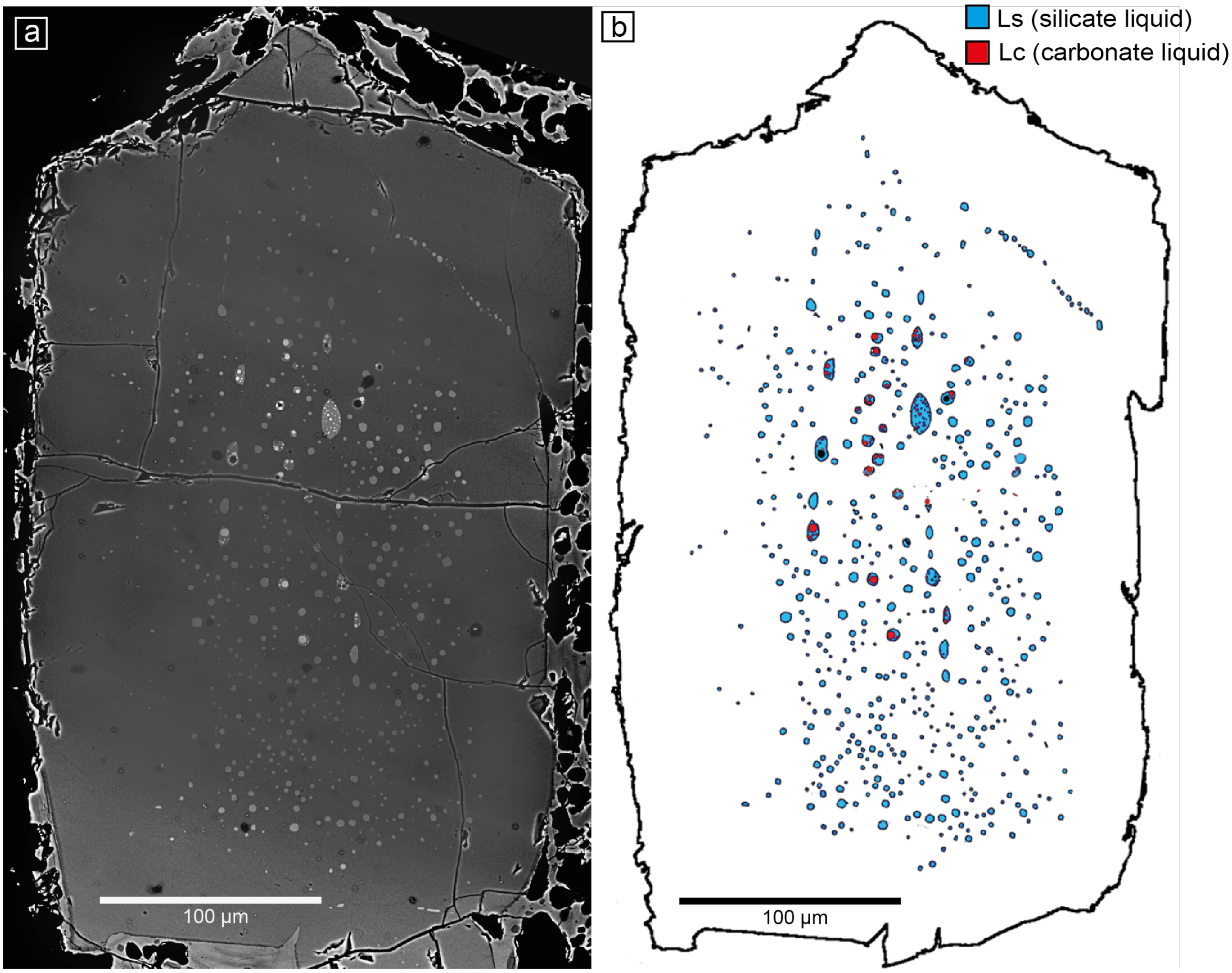


**Supplementary Information Figure 1:** Hauyne (a) grain with detected modal % Ls and Lc (b).

BSE image (a) of an exemplary hauyne grain showing melt inclusions containing immiscible silicate (blue) and carbonate (red) liquids (Figure b).

**Composition of entrapped phonolite melt parental to Ls and Lc**

While phonolites often occur in spatial relations with carbonatites[1,2], the exact composition of the phonolite being parental to Ls and Lc in our study is unclear. According to Liebsch[3] and Schmitt et al.[4] melt from the middle part of the magma chamber, namely layer 1034 from which investigated samples have been collected, represent the potential parental phonolitic melt that has separated to Ls and Lc. However, as shown in Figure 3, the bulk rock composition of layer 1034[5] slightly deviates from the parental melt, mainly in terms of $SiO_2$, $Al_2O_3$, and CaO. Thus, the entrapped parental melt must have been a slightly more primitive phonolitic liquid. This is confirmed by a calculated estimation (Lp, Table 1) from the compositions and modal abundances of Ls, Lc, and Ls* indicating that the melt hosting hauyne crystals must have crystallized in less evolved parts of the magma chamber.

**Further constraints on liquid immiscibility mechanisms, $H_2O$ and $CO_2$ concentrations in Lc and Ls, and p-T conditions for carbonatite genesis**

Different quenched stages of silicate-carbonate melt separation depending on actual size and shape of the melt inclusions and hence varying thermal gradients during cooling are shown in Figure 2. Assuming the melt inclusions were of similar composition at the time of entrapment and had separated into Ls-Lc liquids before eruption, no such variation in separation stages would occur. More likely, Ls-Lc separation in this part of the magma chamber took place as the LST erupted and the entrapped phonolite liquid fell below the critical solution temperature where the binodal ("solvus") of the silicate-carbonate system was crossed. The melt became unstable and liquid immiscibility took place via binodal demixing/nucleation and growth as indicated by the typical pattern of separate nuclei formation (Figure 2) (e.g.[6,7]). Although cooling must have been quick, probably within seconds to minutes, no spinodal decomposition took place being typical for fast quenched immiscible systems[8]. Thus, liquid immiscibility via binodal demixing/nucleation and growth may be the dominating process for many silicate-carbonate systems as cooling is usually much slower in most geological carbonatite settings, and crossing the metastable region between binodal and spinodal without nucleation is unlikely.

The average analytical total of Lc with C measured is 97.8$\pm$2.1 wt% vaguely indicating lower $H_2O$ contents in carbonate liquids compared to Ls where totals indicate $H_2O$ concentrations of about 3.3$\pm$1.9 wt% $H_2O$. However, this is in good agreement with SIMS-determined $H_2O$ contents in LST melt inclusions of 1.5–5.5 wt%[9] and experimentally confined Laacher See phonolite magma water concentrations around 5–6 wt%[10,11]. $CO_2$ concentrations of LST whole rocks have been determined by Wörner and Schmincke[5] ranging between 100 and 500 ppm which is in overall agreement with Behrens et al.[12] who reported $CO_2$ solubilities in water-bearing phono-tephritic melt compositions at 200 MPa and 1250°C of 800 ppm at 4 wt% $H_2O$. Thus, no $CO_2$, if analyzed, was detected in Ls as the average EPMA $CO_2$ detection limit in this study was ~ 0.34 wt% (3$\sigma$).

Experimental studies reveal that liquid immiscibility is favored at crustal pressures ($\leq$0.1-1GPa[13–15]) due to higher $P_{CO_2}$[13] while temperatures range from 1250°C for simple analog $SiO_2$-$Na_2O$-$Al_2O_3$-CaO-$CO_2$ systems[13]

to 700°C for carbonated natural nephelinite[15]. The experimental data agree well with Laacher See magma chamber pressures of ~200 MPa (and less during eruption) at temperatures of ≤760°C[10].

**References**


1. Mitchell, R. H. Carbonatites and Carbonatites and Carbonatites. *Can. Mineral.* **43**, 2049–2068 (2005).
2. Woolley, A. R. & Kjarsgaard, B. A. Carbonatite occurrences of the world: map and database. 5796 (2008)
3. Liebsch, H. Die Genese der Laacher See-Karbonatite. (Georg-August-Universität Göttingen, 1997).
4. Schmitt, A. K., Wetzel, F., Cooper, K. M., Zou, H. & Wörner, G. Magmatic Longevity of Laacher See Volcano (Eifel, Germany) Indicated by U–Th Dating of Intrusive Carbonatites. *J. Petrol.* **51**, 1053–1085 (2010).
5. Wörner, G. & Schmincke, H.-U. Mineralogical and Chemical Zonation of the Laacher See Tephra Sequence (East Eifel, W. Germany). *J. Petrol.* **25**, 805–835 (1984).
6. Garcia-Ojalvo, J., Lacasta, A. M., Sancho, J. M. & Toral, R. Phase Separation Driven by External Fluctuations. *Europhys. Lett. EPL* **42**, 125–130 (1998).
7. Gebauer, D., Kellermeier, M., Gale, J. D., Bergström, L. & Cölfen, H. Pre-nucleation clusters as solute precursors in crystallisation. *Chem Soc Rev* **43**, 2348–2371 (2014).
8. Cahn, J. W. On spinodal decomposition. *Acta Metall.* **9**, 795–801 (1961).
9. Harms, E. & Schmincke, H.-U. Volatile composition of the phonolitic Laacher See magma (12,900 yr BP): implications for syn-eruptive degassing of S, F, Cl and $H_2O$. *Contrib. Mineral. Petrol.* **138**, 84–98 (2000).
10. Berndt, J., Holtz, F. & Koepke, J. Experimental constraints on storage conditions in the chemically zoned phonolitic magma chamber of the Laacher See volcano. *Contrib. Mineral. Petrol.* **140**, 469–486 (2001).
11. Harms, E., Gardner, J. E. & Schmincke, H.-U. Phase equilibria of the Lower Laacher See Tephra (East Eifel, Germany): constraints on pre-eruptive storage conditions of a phonolitic magma reservoir. *J. Volcanol. Geotherm. Res.* **134**, 125–138 (2004).
12. Behrens, H. *et al.* Solubility of $H_2O$ and $CO_2$ in ultrapotassic melts at 1200 and 1250°C and pressure from 50 to 500 MPa. *Am. Mineral.* **94**, 105–120 (2009).
13. Brooker, R. A. & Kjarsgaard, B. A. Silicate–Carbonate Liquid Immiscibility and Phase Relations in the System $SiO_2$–$Na_2O$–$Al_2O_3$–CaO–$CO_2$ at 0·1–2·5 GPa with Applications to Carbonatite Genesis. *J. Petrol.* **52**, 1281–1305 (2011).
14. Kjarsgaard, B. A. Phase relations of a Carbonated High-CaO Nephelinite at 0.2 and 0.5 GPa. *J. Petrol.* **39**, 2061–2075 (1998).
15. Kjarsgaard, B. A., Hamilton, D. L. & Peterson, T. D. Peralkaline Nephelinite/Carbonatite Liquid Immiscibility: Comparison of Phase Compositions in Experiments and Natural Lavas from Oldoinyo Lengai. in *Carbonatite Volcanism: Oldoinyo Lengai and the Petrogenesis of Natrocarbonatites* (eds. Bell, K. & Keller, J.) 163–190 (Springer Berlin Heidelberg, 1995).
