# Supplementary material for: Origin of carbonatites—liquid immiscibility caught in the act
Source: Nat Commun. 2022 May 24;13:2892. doi: 10.1038/s41467-022-30500-7 (PMC9130134; doi:10.1038/s41467-022-30500-7)
Supplement: Supplementary file 2 — Description of Additional Supplementary Files [file 41467_2022_30500_MOESM2_ESM.pdf]

File Name: Supplementary Data 1
Description: Electron microprobe melt inclusion analyses of conjugate carbonate-silicate liquids and single phonolitic and carbonate melts
